# Retrospective observational study of a novel smartphone app on the management of patients with mild cognitive impairment or mild dementia

**DOI:** 10.3389/fdgth.2023.1243253

**Published:** 2023-09-12

**Authors:** Reo Hamaguchi, Yoshihiro Hirokawa, Hirotsugu Takahashi, Tsuyoshi Hachiya, Haruna Kawano, Shuji Isotani, Emi Ito, Nobuhiro Handa, Ryozo Saito, Shigeo Horie, Hisamitsu Ide

**Affiliations:** ^1^ Department of Digital Therapeutics, Graduate School of Medicine, Juntendo University, Tokyo, Japan; ^2^ Hirokawa Clinic, Kyoto, Japan; ^3^ Department of Advanced Informatics for Genetic Disease, Graduate School of Medicine, Juntendo University, Tokyo, Japan; ^4^ Department of Urology, Graduate School of Medicine, Juntendo University, Tokyo, Japan; ^5^ Life Quest Inc., Tokyo, Japan

**Keywords:** smartphone app, digital therapy, nonpharmacological intervention, mild cognitive impairment, dementia

## Abstract

**Introduction:**

In this study, we aimed to evaluate the feasibility, utility, and potential effects of LQ-M/D App, a smartphone application developed by Life Quest Inc., Tokyo, Japan, for patients with mild cognitive impairment (MCI) and mild dementia. The app incorporates cognitive and physical exercise training, lifestyle habit acquisition features, and a continuity improvement feature added in the post-update version to enhance user engagement. The continuity improvement feature includes the optimization of training content, and disease education, and enables family monitoring via a family app.

**Methods:**

A retrospective analysis was conducted on app usage, cognitive and exercise training implementation and interruptions, questionnaire response rates, and cognitive assessments in a single institution. A total of 20 patients used the app, with 10 patients using the pre-update version without the continuity improvement feature, and the other 10 patients using the post-update version with the continuity improvement feature.

**Results and Conclusion:**

The results demonstrated that the LQ-M/D App could be effectively used by the study population, and the continuity improvement feature positively influenced app usage in several aspects. Although a potential association between app usage and cognitive ability was suggested, the scatter in the data points warrants cautious interpretation. Limitations of the study included a small sample size, a single institution setting, and the retrospective nature of the study. In the future, a randomized controlled trial design using a larger sample size and multiple institutions to further evaluate the effectiveness of LQ-M/D App in managing MCI and mild dementia should be performed.

## Introduction

The prevalence of dementia in Japan is increasing, and the number of people in Japan with dementia in 2025 is estimated to be 6.75 million (1). Although pharmacotherapy is thought to alleviate symptoms in patients who have developed dementia, its effectiveness in halting or slowing the progression of the disease is very limited. Therefore, interventions are recommended to be performed in patients with mild cognitive impairment (MCI) before the onset of dementia or in the early stages of dementia. The prevalence of MCI is reported to be 15%–25% in those aged 65 years and older (2–4), and the conversion rate from MCI to dementia is reported to be approximately 5%–15% per year (5). On the other hand, MCI-to-normal reversion has also been reported to be 16%–41% per year (6), and although it is controversial whether such improvement can actually be observed, it is considered important to actively address this from the time a patient has MCI, to prevent subsequent dementia. Cholinesterase inhibitors used for dementia have been reported to improve cognitive function in MCI patients; however, they have not been shown to prevent the progression to dementia (7). Non-drug therapies centered on moderate physical activity and lifestyle modification have been recommended to reduce the progression from MCI to dementia (8–10). In addition, lifestyle-associated diseases, such as hypertension, diabetes, dyslipidemia, and a history of cerebrovascular disease are known to be risk factors for the progression from MCI to dementia, and appropriate management of these risk factors is important (10, 11). Management of these risk factors requires that the patient and family members have an appropriate understanding of MCI, and they receive guidance to prepare for the progression to dementia, including understanding and improving lifestyle habits.

In recent years, the field of mobile health using smartphones and mobile phones has gained attention, and is beginning to be used for older adults (12, 13), including those with MCI (14, 15). However, treatment-focused apps designed specifically for patients with MCI or dementia are limited at present. Cognitive Assessment for Dementia, iPad version (CADi), and the Cogstate Brief Battery (CBB) are examples of apps intended for the assessment of cognitive function (16, 17). CADi can be used as a new mass screening tool that comprises memory and cognitive tasks, and has demonstrated good reliability and validity for the screening of patients with dementia (16). CBB, on the other hand, has shown promise for unsupervised at-home cognitive assessments, but it primarily focuses on evaluation, rather than intervention (17). Exercise-based interventions are expected to slow cognitive decline as non-pharmacological therapies (18), and it was reported that a smartphone app called “HealtheBrain” can be used without any problems in older adults with or without MCI (19). This app, designed to provide a visuospatial memory exercise called the square stepping exercise, aims to improve cognitive function in older adults. In addition, cognitive training, traditionally conducted on paper but now often conducted using computers, has been suggested to be effective for cognitive function (20). A digital calendar with activity/event reminders via short messages that can be used on smartphones or mobile phones, which can be set by family members, professionals, or other supporters, has been used by older adults aged 65 years and older (including older adults who are aware of memory loss), and it was reported that the use of this system can be learned easily (21).

As described above, it has been reported that providing exercises, scheduling, and obtaining activity records via smartphones can be used without problems by older patients, including those with cognitive impairment. In addition, computer-based cognitive training can be substituted by mobile devices, such as smartphones. A smartphone application for patients with MCI or mild dementia called “LQ-M/D App” was developed by Life Quest Inc. (Tokyo, Japan), which includes the following functions: (i) training content (cognitive training and exercise training), and (ii) content for the acquisition of lifestyle habits (diet, sleep, and walking time). At present, there are a limited number of treatment-focused apps specifically tailored for patients with MCI or dementia. The LQ-M/D App is specifically designed for older adults with cognitive impairments to provide a non-pharmacological intervention. The purpose of this study was to investigate the feasibility and utility of the LQ-M/D App, and we conducted a retrospective observational study to analyze the clinical and application data of patients with MCI or mild dementia who used LQ-M/D App.

## Patients and methods

### Study design

This observational study was retrospectively conducted to investigate the effects of LQ-M/D App on patients with MCI or mild dementia treated at Hirokawa Clinic, Kyoto, Japan, from September 1, 2020, to August 31, 2021, using the clinic's medical records and data from the app. All patients were provided with smartphones containing the pre-downloaded LQ-M/D App, and used the LQ-M/D App, as well as received standard treatments for MCI or mild dementia. All procedures were performed in accordance with the ethical principles described in the 1995 Declaration of Helsinki. Written informed consent was obtained from each patient. The study was approved by the Institutional Review Board of Juntendo University and registered in UMIN Clinical Trials (UMIN000047077). In this study, external monitoring by the Japan Organization for Research and Treatment of Cancer (JORTC) verified data accuracy by comparing original sources with analysis data for a random subset of cases, with no discrepancies identified, confirming data integrity.

### LQ-M/D App

**Figure 1:**
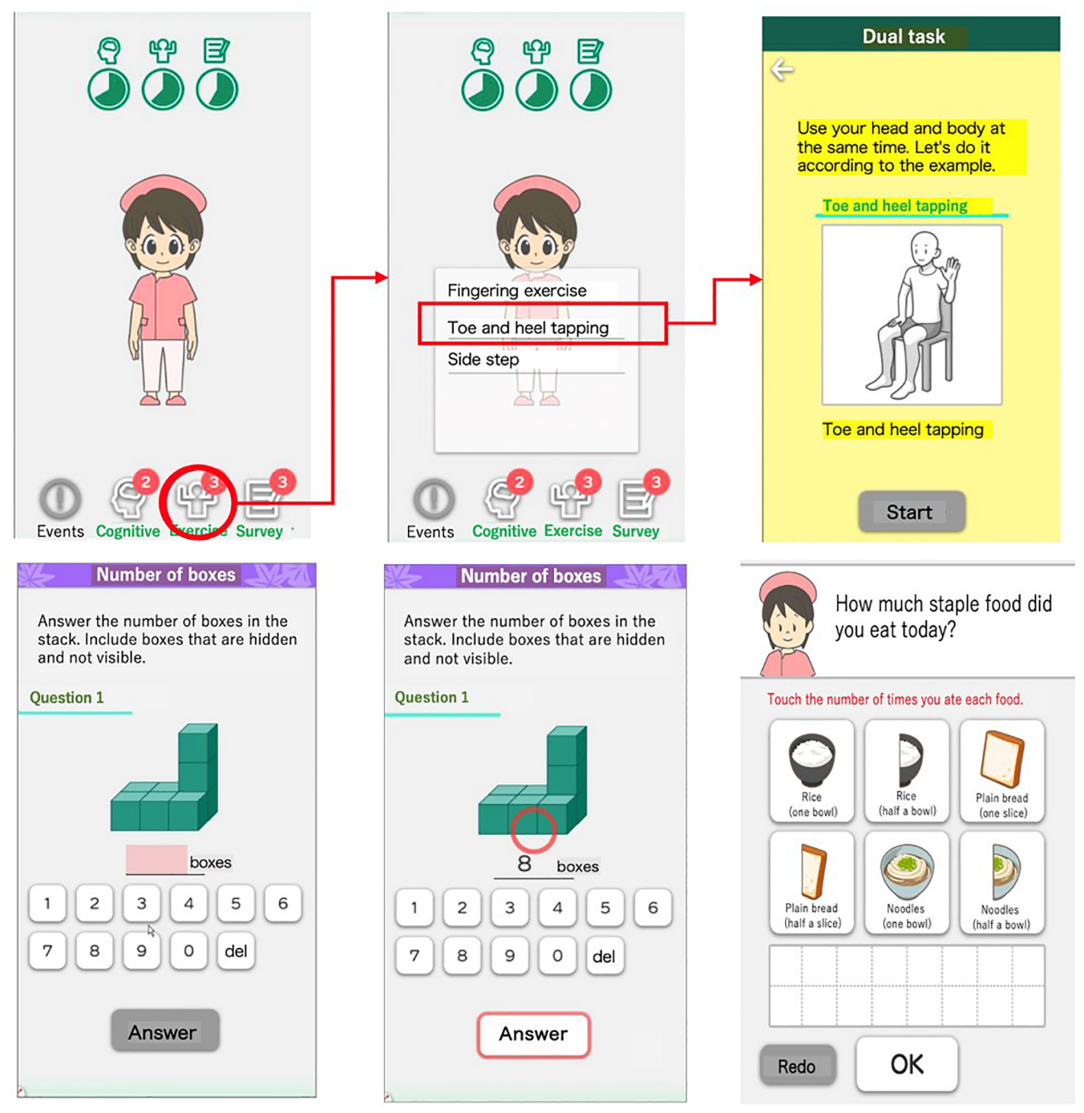
Overview of LQ-M/D App main features. Screenshots of LQ-M/D App, showcasing its main features: (i) training content, and (ii) acquisition of lifestyle habits. Images depict examples of exercise training (top-right), cognitive training (bottom-left and center-bottom), and the dietary questionnaire (bottom-right).

In this study, the smartphone application “LQ-M/D App” developed by Life Quest Inc. was used. LQ-M/D App has two main features: (i) provision of training content, and (ii) content for the acquisition of lifestyle habits (Figure 1).

The training content consists of cognitive function training and physical exercise training. The cognitive training consists of multiple menus targeting various cognitive functions, including memory function, language function, judgment function, calculation function, executive function, and visuospatial cognitive function, with a total of 21 different tasks. The physical exercise training comprises dual-task training and other types of training, and includes 38 dual-task exercises and 24 other types of physical activities. The lifestyle habits acquisition feature collects information on diet, sleep, and walking time through questionnaires conducted within the app. However, at present, the app does not have a feature for physicians to review records and provide feedback.

**Figure 2:**
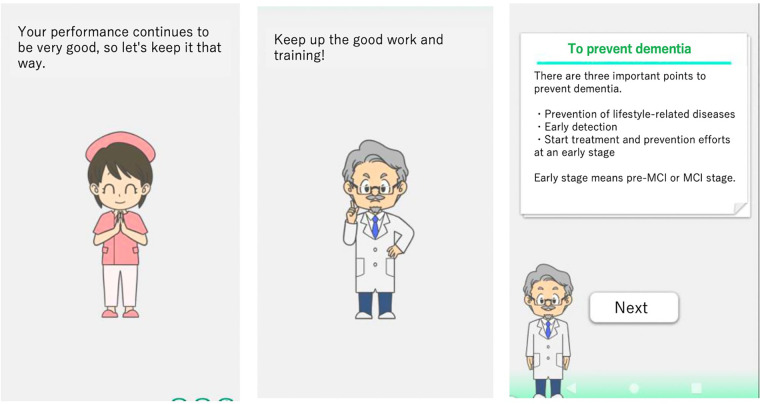
Enhancements in the post-updated LQ-M/D App. Screenshots of the post-updated LQ-M/D App, highlighting continuity improvement features. The left and center images show avatar interventions, and the right image presents educational content. These features aim to enhance patient engagement and understanding.

On December 21, 2020, LQ-M/D App was updated to include a continuity improvement feature designed to enhance sustained user engagement. This feature consists of optimizing training content, providing disease education, and enabling family monitoring via a family app (Figure 2). The optimization of training content adjusts and reconfigures the content based on usage, with varying intervention frequencies by an avatar. Disease education offers valuable information and resources to help patients and their families better understand and manage the condition. The family app enables monitoring of the patient's app usage, and enables simple message exchanges between patients and their family members. As a result, families can better understand the patient's situation, and an improvement in the patient's app usage can be expected. A summary of the features of the LQ-M/D App is shown in Table 1.

**Table 1:** Summary of features of the LQ-M/D App.

Feature	Pre-update version	Post-update version
Training content		
Cognitive function training^a^	○	○
Physical exercise training^a^	○	○
Lifestyle habits acquisition		
Collection of information on diet, sleep, and daily walking time^a^	○	○
Continuity improvement feature		
Optimizing training content^a^	×	○
Providing disease education^a^	×	○
Family monitoring via family app^b^	×	○

^a^ All features except for the family monitoring feature are utilized by patients through the LQ-M/D App.

^b^ The family monitoring feature is utilized by family members through the family app.

In this study, we used both the pre-update version without the continuity improvement feature, and the post-update version with the continuity improvement feature, each for a duration of 6 months, to compare and investigate their respective effects. Patients and caregivers were provided with comprehensive instructions on how to use the app. During regular monthly check-ups, healthcare providers asked about app usage to evaluate its use by the patients and caregivers. However, the actual usage of the app was ultimately at the discretion of the users themselves.

### Assessment procedures

In this study, data from medical records were retrospectively collected, including patient background, MCI and dementia treatment courses, and scores for Mini-Mental State Examination (MMSE), Hasegawa's Dementia Scale-Revised (HDS-R), Clinical Dementia Rating (CDR), and Alzheimer's Disease Assessment Scale-Cognitive Subscale Japanese version (ADAS-jcog). Additionally, information on app malfunctions occurring during use, daily app usage duration (in minutes), the daily number of cognitive training and exercise training sessions performed, daily number of interruptions of cognitive training and exercise training, and the presence or absence of daily questionnaire responses were obtained from the app data. The data were compared between the pre-update version without the continuity improvement feature and the post-update version with the continuity improvement feature. Furthermore, MMSE scores of 24–27 points were classified as MCI, and those of 20–23 points as mild dementia. For these patients, the association between daily app usage duration (in minutes), the daily number of cognitive training and exercise training sessions, and changes in MMSE, HDS-R, and ADAS-jcog scores from the start of app use to 6 months after the end of app usage were examined.

### Statistical analyses

For both the pre-update version without the continuity improvement feature and the post-update version with the continuity improvement feature, unpaired *t*-tests were conducted for daily app usage duration (in minutes), daily number of cognitive training and exercise training sessions, and daily number of interruptions for cognitive training and exercise training. Additionally, the Mann-Whitney *U*-test was performed to compare the presence or absence of daily questionnaire responses between the pre-update and post-update versions. Pearson's correlation coefficients were measured between the changes in MMSE, HDS-R, and ADAS-jcog scores (6-month score—initial score) and daily app usage duration, and daily number of cognitive training and exercise training sessions. Standard deviations of mean dataset values were calculated. All *p*-values were two-sided, and a *p*-value of less than 0.05 was considered to indicate a statistically significant difference between two groups. All statistical analyses were performed using Easy R software (version 1.61; Saitama Medical Center, Jichi Medical University, Saitama, Japan), a graphical user interface that is a modified version of R (The R Foundation for Statistical Computing, Vienna, Austria) (22).

## Results

### Patient characteristics

A total of 20 patients (7 men and 13 women) used LQ-M/D App, with a mean age of 72.3 (range: 50–82) years at the start of app usage. The MMSE, HDS-R, CDR, and ADAS-jcog scores at the start of app usage are shown in Table 2. The mean daily app usage duration (minutes), mean daily number of cognitive training and exercise training sessions, mean daily number of interruptions, and the proportion of questionnaire responses are presented in Table 3 for the entire study period as well as from the first month to the sixth month.

**Table 2:** Patient characteristics.

Number of patients	20
Age, years	
Mean (range)	72.3 (50–82)
Sex	
Men	7
Women	13
MMSE score	24.7 ± 3.0
HDS-R score	24.4 ± 3.7
CDR score	0.45 ± 0.15
CDR category (number of patients)	
0	2
0.5	18
1	0
2	0
ADAS-jcog score	10.4 ± 3.8
Medication (anti-dementia drugs)	
Yes	17
No	3

MMSE, mini-mental state examination; HDS-R, Hasegawa's dementia scale-revised; CDR, clinical dementia rating; ADAS-jcog, Alzheimer's disease assessment scale-cognitive subscale Japanese version.

**Table 3:** Summary of App usage, training sessions, interruptions, and response rates.

	App usage duration (minutes/day)	Cognitive training		Exercise training		Questionnaire
		Sessions (times/day)	Interruptions (times/day)	Sessions (times/day)	Interruptions (times/day)	Response rate per day (%)
Observation period (6 months)	38.2 ± 41	7.6 ± 10.7	0.125 ± 0.420	3.8 ± 5.3	0.159 ± 0.517	21.7 ± 42.1
1st month	44.9 ± 39.5	8.2 ± 9.3	0.152 ± 0.489	4.0 ± 4.7	0.249 ± 0.784	22.4 ± 44.1
2nd month	39.1 ± 45.7	6.6 ± 9.5	0.118 ± 0.382	4.5 ± 7.0	0.200 ± 0.589	23.8 ± 42.9
3rd month	42.1 ± 46.8	8.9 ± 13.1	0.134 ± 0.390	4.5 ± 5.6	0.158 ± 0.420	23.4 ± 43.9
4th month	34.9 ± 40.4	7.6 ± 12.3	0.135 ± 0.446	3.2 ± 4.8	0.123 ± 0.373	19.6 ± 41.5
5th month	35.7 ± 35.9	7.8 ± 10.0	0.100 ± 0.386	3.5 ± 4.8	0.113 ± 0.354	21.6 ± 40.8
6th month	29.3 ± 31.1	6.3 ± 8.5	0.100 ± 0.400	2.8 ± 4.0	0.077 ± 0.292	17.9 ± 37.0

Data are shown as mean ± standard deviation.

### App malfunctions

During usage of the app, 11 patients experienced malfunctions. In 4 cases of patients using the pre-updated version, the training feature of the app was unstable for 36, 34, 26, and 8 days, respectively. In 6 cases of patients using the post-updated version, the app could not be launched owing to an inability to communicate with the server for 14, 13, 12, 8, 6, 5, and 4 days, respectively.

### Comparison between the pre-update version and the post-update version

**Figure 3:**
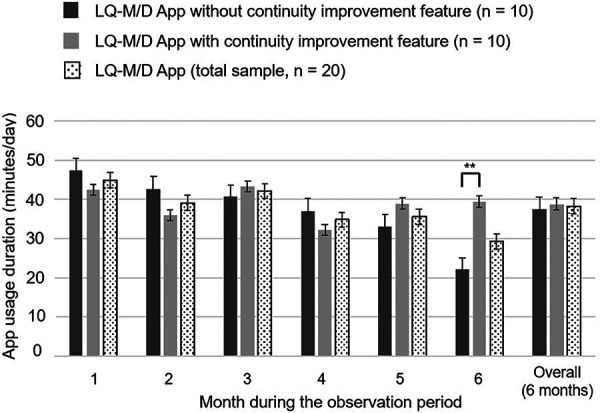
LQ-M/D App usage duration. Usage duration of LQ-M/D App (*n* = 20), LQ-M/D App without the continuity improvement feature (*n* = 10), and LQ-M/D App with the continuity improvement feature (*n* = 10), from the first to the sixth month, as well as the entire observation period (6 months). The graph shows the mean values for app usage duration in each group, with error bars representing the standard error. *p < 0.05, **p < 0.005.

**Figure 4:**
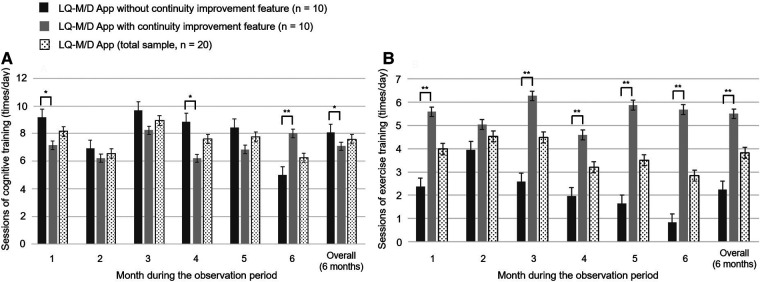
Number of cognitive and exercise training sessions on LQ-M/D App. Number of sessions of cognitive and exercise training performed by patients who used LQ-M/D App (*n* = 20), LQ-M/D App without the continuity improvement feature (*n* = 10), and LQ-M/D App with the continuity improvement feature (*n* = 10), from the first to the sixth month, as well as the entire observation period (6 months). (**A**) Graph showing the mean values for sessions of cognitive training performed by each group, with error bars representing the standard error. (**B**) Graph showing the mean values for sessions of exercise training performed by each group, with error bars representing the standard error. *p < 0.05, **p < 0.005.

**Figure 5:**
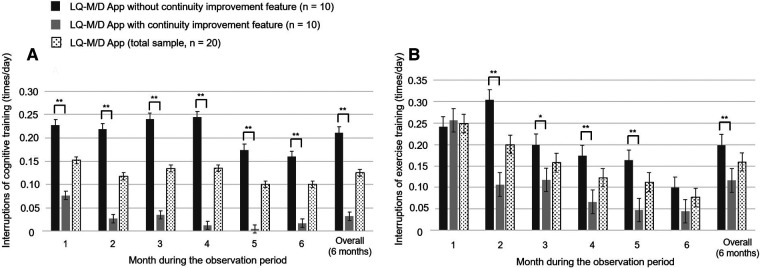
Interruptions in cognitive and exercise training on LQ-M/D App. Interruptions of cognitive and exercise training of patients who used LQ-M/D App (*n* = 20), LQ-M/D App without the continuity improvement feature (*n* = 10), and LQ-M/D App with the continuity improvement feature (*n* = 10), from the first to the sixth month, as well as for the entire observation period (6 months). (**A**) Graph showing the mean values for interruptions of cognitive training in each group, with error bars representing the standard error. (**B**) Graph showing the mean values for interruptions of exercise training in each group, with error bars representing the standard error. *p < 0.05, **p < 0.005.

**Figure 6:**
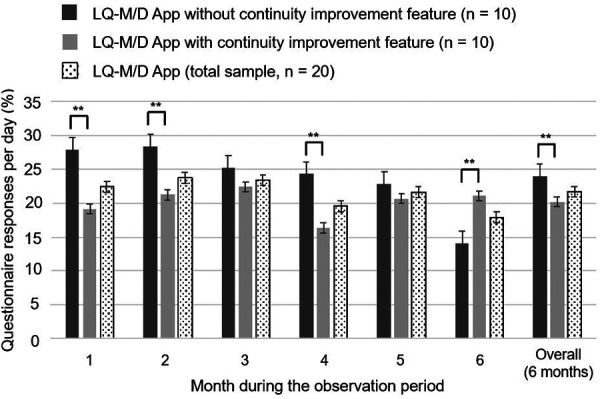
Questionnaire response rates on LQ-M/D App. Questionnaire responses for LQ-M/D App (*n* = 20), LQ-M/D App without the continuity improvement feature (*n* = 10), and LQ-M/D App with the continuity improvement feature (*n* = 10), from the first to the sixth month, as well as for the entire observation period (6 months). The graph shows the mean values for questionnaire response rates in each group, with error bars representing the standard error. *p < 0.05, **p < 0.005.

In this analysis, the pre-update version without the continuity improvement feature (*n* = 10) was compared with the post-update version with the continuity improvement feature (*n* = 10). For each version, the mean daily app usage time, mean number of cognitive training and exercise training sessions, mean number of interruptions, and presence or absence of questionnaire responses were shown for the entire period and from the first to the sixth month, in Figures 3–6.

Over the entire 6-month observation period, there was no statistically significant difference in mean daily app usage time between the pre-update and post-update versions. Examining the monthly results, the mean usage time tended to decrease over time in the pre-update version, whereas the post-update version did not show a clear decline in mean usage time, and at 6 months, the mean daily app usage time was significantly longer for the post-update version (Figure 3).

Regarding the mean number of daily cognitive training sessions, there was a tendency for the pre-update version to have more sessions, with a significantly higher number of sessions for the overall observation period (6 months), as well as for the first and fourth months. On the other hand, at 6 months, the number of sessions decreased in the pre-update version, and a significant increase in the number of sessions was observed in the post-update version (Figure 4A). For the mean number of interruptions, a statistically significant difference was observed between the pre-update and post-update versions for the overall observation period (6 months) and in each month, with more interruptions in the pre-update version (Figure 5A). The mean number of daily exercise training sessions was significantly higher for the post-update version during the overall observation period of 6 months and in each month, except for the second month (Figure 4B). Additionally, the mean number of interruptions was lower for the post-update version, with a statistically significant difference observed for the overall observation period of 6 months and each month, except for the first and 6 months (Figure 5B).

The mean daily questionnaire response rate tended to be lower for the post-update version, except for the sixth month. A significantly higher mean questionnaire response rate was observed for the pre-update version during the overall 6-month observation period, as well as at the first, second, and fourth months (Figure 6). However, it is difficult to compare the data, as the number of questionnaires differed between the two versions; i.e., in the pre-update version, questionnaires were conducted after breakfast, lunch, and dinner, whereas in the post-update version, questionnaires were additionally conducted upon waking and before bedtime.

### Association between scores for MMSE, HDS-R, and ADAS-jcog, and app usage

**Figure 7:**
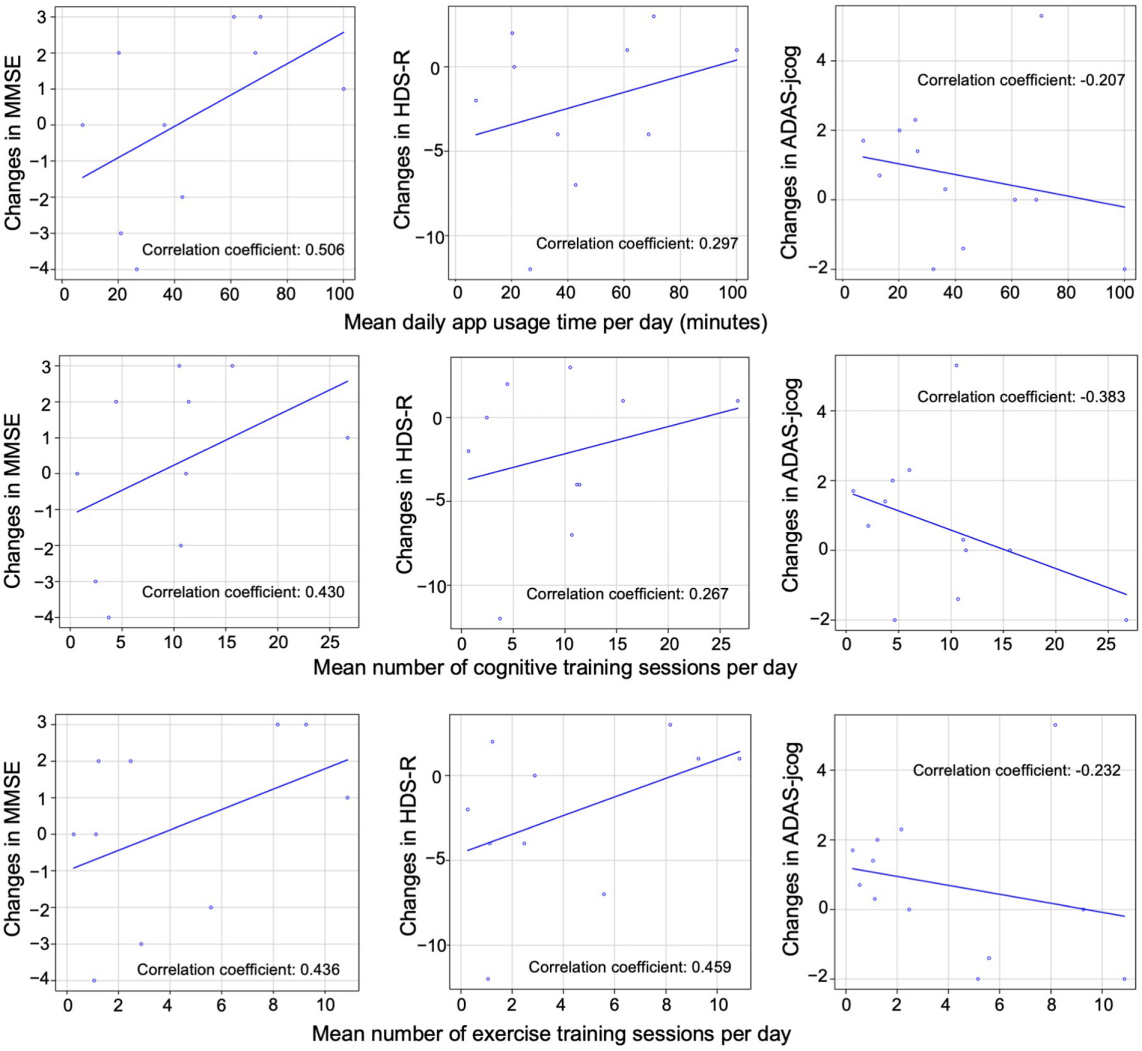
Correlation between app usage and cognitive test scores. Scatterplots illustrating the association between daily app usage duration, daily number of cognitive training sessions, and exercise training sessions with changes in MMSE, HDS-R, and ADAS-jcog scores from the start of app use to 6 months after the end of app use in 15 patients. The Pearson's correlation coefficient for each association is provided. No statistically significant differences were observed between the groups.

In this study, patients with MMSE scores of 24–27 were classified as having mild MCI, and those with scores of 20–23 were classified as having mild dementia. Four patients with MMSE scores of 28 or higher and 1 patient with a score of 19 or lower were excluded. The remaining sample comprised 11 patients with MCI and 4 with mild dementia, totaling 15 patients. The association between daily app usage duration (in minutes), the daily number of cognitive training and exercise training sessions, and changes in MMSE, HDS-R, and ADAS-jcog scores from the start of app use to 6 months after the end of app use was investigated for these 15 patients using Pearson's correlation coefficients. Scatterplots of the associations are shown in Figure 7. The results indicate positive correlations between MMSE and HDS-R cognitive assessments and factors, such as app usage duration, cognitive training sessions, and exercise training sessions, suggesting potential cognitive improvement. In contrast, a negative correlation was observed with ADAS-jcog scores, also implying cognitive enhancement. However, the *p*-values were not statistically significant, suggesting that further research is needed to confirm these findings and their effects on cognitive function.

## Discussion

In this study, we investigated the feasibility and utility of LQ-M/D App, a smartphone application developed for patients with MCI or mild dementia. The results demonstrated that the app could be utilized by the study population without major issues, and that the continuity improvement feature introduced in the updated version of the app appeared to positively influence app usage in several aspects. Although some app malfunctions were observed during the study, they were within acceptable limits and did not greatly hinder the app's usability. However, although the association between app usage and cognitive ability of the patients showed a tendency for a positive correlation, the available data was not sufficient to draw definitive conclusions. Therefore, further research is needed to confirm these findings and their effects on cognitive function.

Previous studies have reported the potential benefits of nonpharmacological interventions, such as moderate physical activity and lifestyle modifications, in slowing cognitive decline and reducing the progression from MCI to dementia (23, 24). LQ-M/D App incorporates cognitive and physical exercise training, as well as lifestyle habit acquisition features, which may contribute to the management of MCI or mild dementia. The app also includes disease education and family monitoring through the family app, which may help patients and their families better understand and manage their condition, in line with recommendations for MCI management (25, 26). The comparison between the pre-update and post-update versions of LQ-M/D App in our study demonstrated that the continuity improvement feature positively influenced app usage duration, the number of cognitive training and exercise training sessions, and the number of interruptions. These findings suggest that the updated app may provide a more engaging and user-friendly experience for patients with MCI or mild dementia, which is consistent with previous reports on the usability of mobile health interventions for older adults (27–29).

Our study observed a generally low questionnaire response rate in both the pre-update and post-update versions of LQ-M/D App. A possible reason for this might be the lack of evaluation and feedback on patients’ questionnaire responses, as feedback has been shown to improve engagement and adherence in eHealth interventions (30). The post-update version showed a lower response rate compared with the pre-update version, potentially owing to response fatigue among patients resulting from the increased frequency of questionnaires in the post-update version (31, 32). Further investigation is needed to determine the optimal questionnaire frequency and the role of feedback in balancing data collection and patient engagement in electronic health interventions.

In this study, the association between app usage and cognitive improvement did not reach statistical significance, with correlation coefficients demonstrating wide-ranging confidence intervals and relatively high *p*-values. These findings suggest a potential association between app usage and cognitive improvement but should be interpreted with caution owing to the small sample size and the retrospective nature of the study. Moreover, among the 20 patients, 17 were receiving medication for dementia, and 2 patients started taking medication during the study period. Therefore, the possibility of the slight improvement in cognitive performance being a result of the effects of the medication rather than the effects of training using the App cannot be ruled out. Further research with larger sample sizes and prospective study designs is necessary to better understand the effects of the LQ-M/D App on cognitive function in patients with MCI or mild dementia, and to confirm the potential cognitive benefits of the app and to investigate the underlying mechanisms contributing to these correlations, as suggested by prior research on the effectiveness of cognitive training for cognitive function (33).

There are some limitations to our study. First, the sample size was relatively small, which may have affected the statistical power of the analyses. Second, the study was conducted in a single institution, which may limit the generalizability of the findings. Third, the study was retrospective in nature, which may have introduced biases in the data collection and interpretation. Fourth, the study lacked a control group, making it difficult to determine the specific effects of the LQ-M/D App on cognitive function compared with standard treatments alone. Future research should consider a randomized controlled trial design with larger sample sizes and multiple institutions to further evaluate the effectiveness of LQ-M/D App in managing MCI and mild dementia.

## Conclusions

In conclusion, this study demonstrated the feasibility and potential utility of LQ-M/D App for patients with MCI or mild dementia. The app's continuity improvement feature appeared to positively influence app usage, and the observed trends in cognitive assessments suggested potential cognitive improvement, although further research is needed to confirm these findings. LQ-M/D App may be a promising tool for the management of MCI and mild dementia, potentially contributing to the prevention or delay of cognitive decline in this population.

## Data Availability

The raw data supporting the conclusions of this article will be made available by the authors, without undue reservation.
